# Construction of cardiac fibrosis for biomedical research

**DOI:** 10.1002/SMMD.20230020

**Published:** 2023-08-16

**Authors:** Yixuan Shang, Rui Liu, Jingjing Gan, Yuzhi Yang, Lingyun Sun

**Affiliations:** ^1^ Department of Medical Supplies Support Nanjing Drum Tower Hospital Affiliated Hospital of Medical School Nanjing University Nanjing China; ^2^ Department of Rheumatology and Immunology Nanjing Drum Tower Hospital Affiliated Hospital of Medical School Nanjing University Nanjing China

**Keywords:** biomedical materials, cardiac fibrosis, drug screening, tissue engineer

## Abstract

Cardiac remodeling is critical for effective tissue recuperation, nevertheless, excessive formation and deposition of extracellular matrix components can result in the onset of cardiac fibrosis. Despite the emergence of novel therapies, there are still no lifelong therapeutic solutions for this issue. Understanding the detrimental cardiac remodeling may aid in the development of innovative treatment strategies to prevent or reverse fibrotic alterations in the heart. Further combining the latest understanding of disease pathogenesis with cardiac tissue engineering has provided the conversion of basic laboratory studies into the therapy of cardiac fibrosis patients as an increasingly viable prospect. This review presents the current main mechanisms and the potential tissue engineering of cardiac fibrosis. Approaches using biomedical materials‐based cardiac constructions are reviewed to consider key issues for simulating in vitro cardiac fibrosis, outlining a future perspective for preclinical applications.

## Graphical abstract

Despite substantial advances in tissue‐engineered cardiac fibrosis model research, there has been limited attention on their features and uses, as well as conceivable development and challenges. This review highlights biomedical material fabrication procedures and the construction of in vitro cardiac fibrosis models, provides a comprehensive overview of the remaining issues, and predicts the future cardiac fibrosis models.

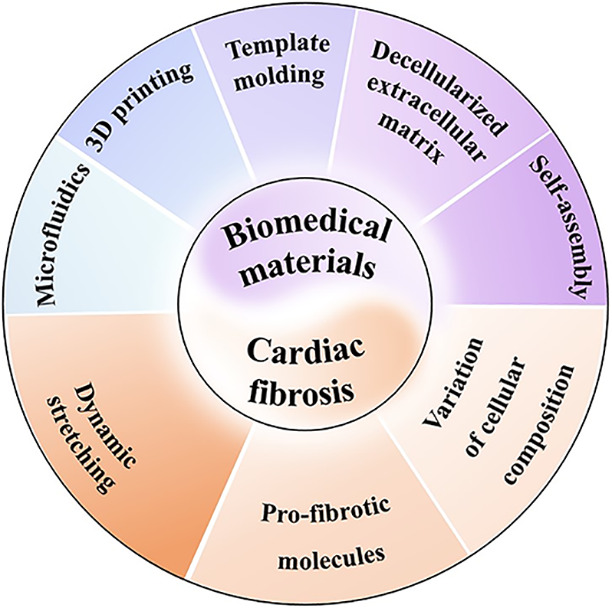

**Key points**

- The fabrication methods of biomedical materials are discussed.
- The novel incorporation between biomedical materials and special structures for in situ tests.
- Introduced the various constructions of cardiac fibrosis with biomedical materials.
- Overviewed the challenges and potential of the current cardiac fibrosis models.

## 1 INTRODUCTION

Heart disease affects over 30% of the global population and is the top cause of death worldwide.^1^, ^2^, ^3^ In addition to changes in myocardial cell structure and cell composition, in various types of cardiomyopathies, cardiac fibrosis usually occurs after adverse ventricular remodeling.^4^, ^5^ Fibrosis has been proven to be a serious negative prognostic indicator in cases of heart failure, which can directly worsen disease outcomes.^4^, ^6^, ^7^ By simulating the physio‐pathological features in vitro, researchers have developed various experimental systems for investigating the mechanisms of cardiac fibrosis.^8^, ^9^, ^10^, ^11^, ^12^ Planar two‐dimensional (2D) culture‐based analysis, such as high‐throughput screening in multi‐well plates, provides image‐based multi‐parameter readout of fibrosis markers.^13^ Owing to marked improvements in stem cell methods, cardiomyocytes originated from induced pluripotent stem cells (iPSC‐CMs) or human embryonic stem cells (hESC‐CMs) have emerged as crucial substitutes for preclinical studies which can not only offer reliable cell sources containing human proteome but also circumvent the species‐dependent differences present in animal models.^14^, ^15^, ^16^, ^17^, ^18^ Additionally, the myocardium hosts multiple cell types, including cardiomyocytes, fibroblasts, nerve cells, endothelial cells, and macrophages.^19^, ^20^ Consequently, numerous research teams have designed multicellular co‐culture models, which enable the precise spatiotemporal manipulation of multiple cell types to mimic cardiac function.^21^, ^22^, ^23^ Notwithstanding significant achievements, there exist pioneering breakthroughs and obstacles in the realm of cardiac fibrosis models. Specifically, extracellular matrix (ECM) components are lacking in these models, which limits the development of tissue‐engineered models capable of recapitulating tissue‐level morphology and physiology.

Many efforts have been made to improve cardiac fibrosis models, with significant emphasis placed on exploring innovative materials and technology.^24^, ^25^ Tissue‐engineered constructs have the potential to serve as a tissue model that can imitate the human biological three‐dimensional (3D) ecosystem owing to their ability to permit the inclusion of native ECM proteins, diverse cells, and essential signaling proteins.^26^, ^27^, ^28^, ^29^ ECM acts as a scaffold for skeletal support and is essential in promoting chemical, electrical, and mechanical cellular contact. Biomedical materials hold great potential as candidates for tissue engineering, which can provide ECM simulants for implanted cells. In particular, materials with micro‐nano architectures have been proved to induce cell orientation and function, thereby mimicking the tissue‐level morphology and enhancing versatility and practicability.^24^ Recently, various microfabrication techniques have been utilized to create ECM simlants that support cell culture and simulate specific disease conditions.^8^, ^30^, ^31^, ^32^, ^33^ Substantial emphasis has been placed on the following exemplary fabrication methods: microfluidics, 3D printing, template molding, decellularized ECM, and self‐assembly. Integrated cardiac models have emerged as an attractive way of mimicking complex and dynamically changing physical environments, thereby enhancing the longevity and reproducibility of cardiac systems in vitro. Especially, these models equipped with special structures possess enhanced functions, such as visual‐stimulus translation and ultra‐sensitivity to force, considerably expanding their uses.^34^, ^35^ Nowadays, versatile cardiac models have become indispensable tools for studying biomechanics and screening therapeutic drugs because of their ability to quantify cardiac contractile, biomechanical, and electrophysiological properties. Thus, by integrating intelligent biomedical materials, cardiac fibrosis models have promising prospects in the emerging precision medicine field.

**FIGURE 1:**
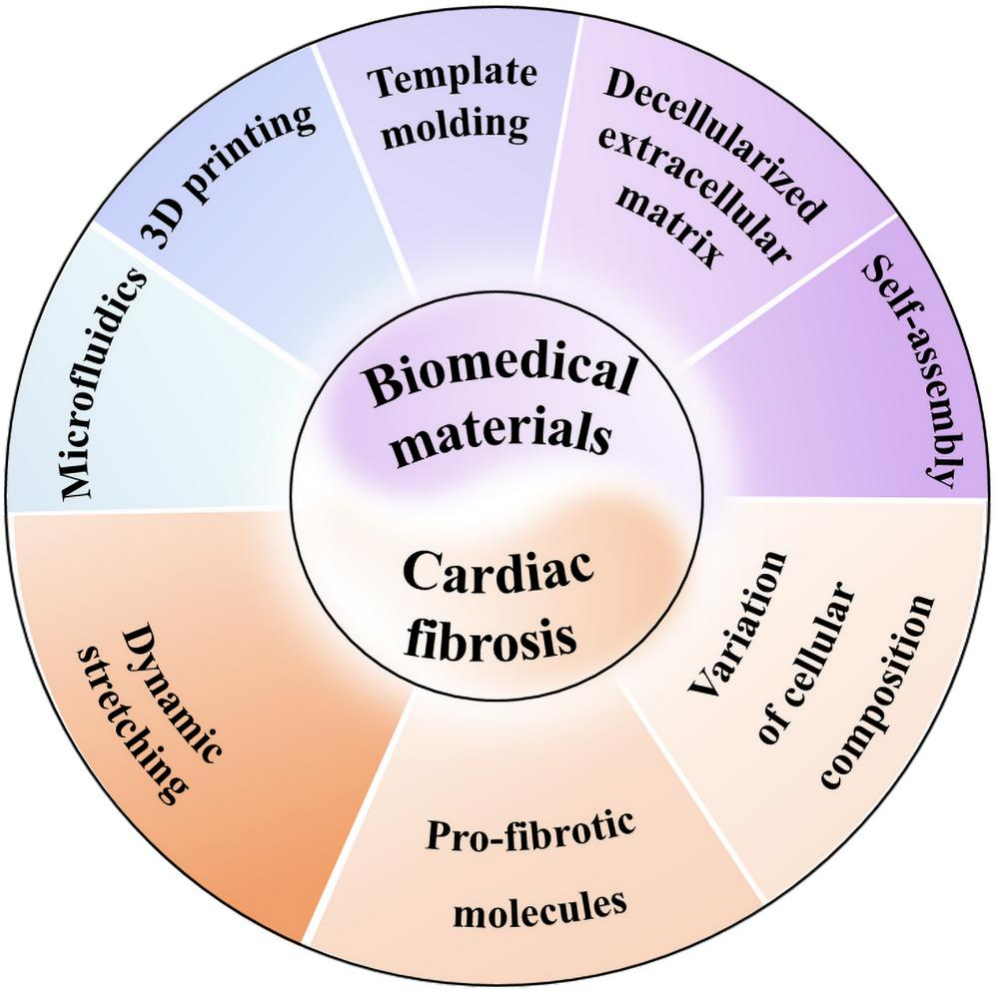
Schematic of biomedical materials fabrication methods and the related cardiac fibrosis construction.

Despite significant achievements in the study of cardiac fibrosis models, limited attention has been paid to their properties and applications of these models, as well as the potential developments and challenges of forthcoming cardiac fibrosis models. In this review, we first introduce biomedical materials and discuss the corresponding fabrication methods. We especially propose the incorporation between biomedical materials and special structures, of which the advantages and the applications for in situ tests are also discussed. Subsequently, we focus on the various constructions of cardiac fibrosis, utilizing representative examples to discuss recent advancements and their corresponding applications (Figure 1). Finally, we provide a comprehensive overview of the lingering issues and offer a forecast for future cardiac fibrosis models.

## 2 FABRICATION OF BIOMIMETIC MATERIALS

The natural ECM acts as a framework for tissue engineering and offers various stimuli to influence cell destiny actions.^36^, ^37^ The biomechanics and physiology of ECMs influence cellular actions, including proliferation, differentiation, and migration.^38^, ^39^ In order to mimic these properties, biomedical materials made of natural carbohydrates, natural and artificial proteins, and manufactured polymers have been employed for cell implantation in 3D culture and transplantation. Particularly, the hierarchical structure of cardiac muscle is highly ordered, and the fibrillar ECM network serves as vital in directing the spatial and temporal synchronicity of cardiac development. Thus, to construct a versatile in vitro heart model, biomedical materials with specialized micro‐nano architectures have been developed. To date, various techniques have been presented to construct ECM simulants, including microfluidics, 3D printing, template molding, decellularized ECM, self‐assembly, and other methods.

### 2.1 Microfluidics

**FIGURE 2:**
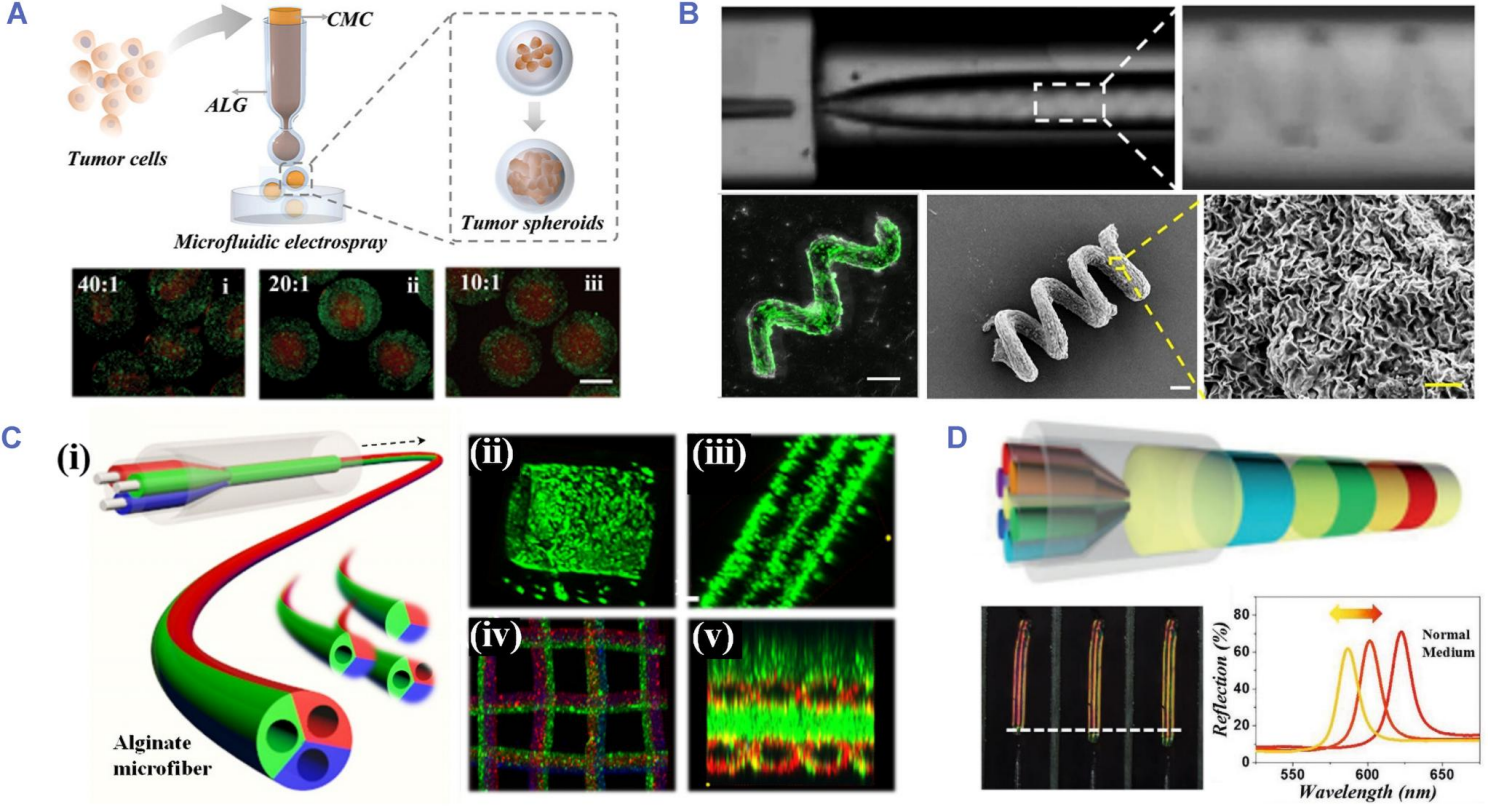
(A) Fabrication of hydrogel microcapsules encapsulated primary human pancreatic cancer cells with a microfluidic electrospray technique. Reproduced under terms of the CC‐BY license.^46^ Copyright 2023, The Authors, published by John Wiley and Sons. (B) Fabrication and cell cultivation of helical microfibers in the microfluidic channel. Reproduced with permission.^47^ Copyright 2020, American Chemical Society. (C) Generation of microfibers and the derived biomimetic vessels and scaffolds. Reproduced with permission.^48^ Copyright 2016, American Chemical Society. (D) Scheme and optical properties of spinning the composite heterogeneous hydrogel microfiber. Reproduced with permission.^51^ Copyright 2021, John Wiley and Sons.

Microfluidic‐based cell encapsulation has gained popularity lately owing to its advantages over alternative microtechnology approaches. Microfluidic methods have permitted the manufacture of high‐throughput, properly controlled monodisperse micrometer‐sized, sphere, or curved microgel components.^40^, ^41^, ^42^, ^43^, ^44^, ^45^ Microfluidic devices may also make microgels in a variety of forms, such as disks, spheres, solid and hollowed fibers, and capsules (core‐shell), which are not conceivable with other fabrication approaches. For example, Song et al. employed a microfluidic electrospray approach to encapsulate primary human pancreatic cancer cells in microcapsules with carboxymethyl cellulose cores and alginate shells (Figure 2A).^46^ With regard to the stability of the technology, accurate architectural stability, and strong monodispersity, the encapsulated cells could proliferate rapidly and dynamically to produce 3D tumor spheroids with extremely uniform size and cell viability. In addition, the straight and helical microfibers could be fabricated by a microfluidic method (Figure 2B,C).^47^, ^48^ These cell‐laden hydrogel microfibers incorporating ECM protein could create a succession of sophisticated 3D constructed architectures by using laminar flow techniques and instantaneous alginate gel formation. With the progress of microfabrication technologies, the biomedical manufacturers of synthetic microfibers have advanced significantly.^49^ Notably, microfibers can be imparted with sensing properties by integrating sensing materials.^50^ Chen and colleagues utilized a programmed injection microfluidic spinning technique to fabricate heterogeneous structural color hydrogel microfibers (Figure 2D).^51^ Through the incorporation of the ordered nanostructures, the resultant heterogeneous structural color hydrogel microfiber could transform microscopic cellar force into amplified identifiable signal. Such a design broadened the scope of biological applications for intelligent fibers, while earlier research concentrated primarily on detecting physical‐chemical variance.

### 2.2 3D printing

**FIGURE 3:**
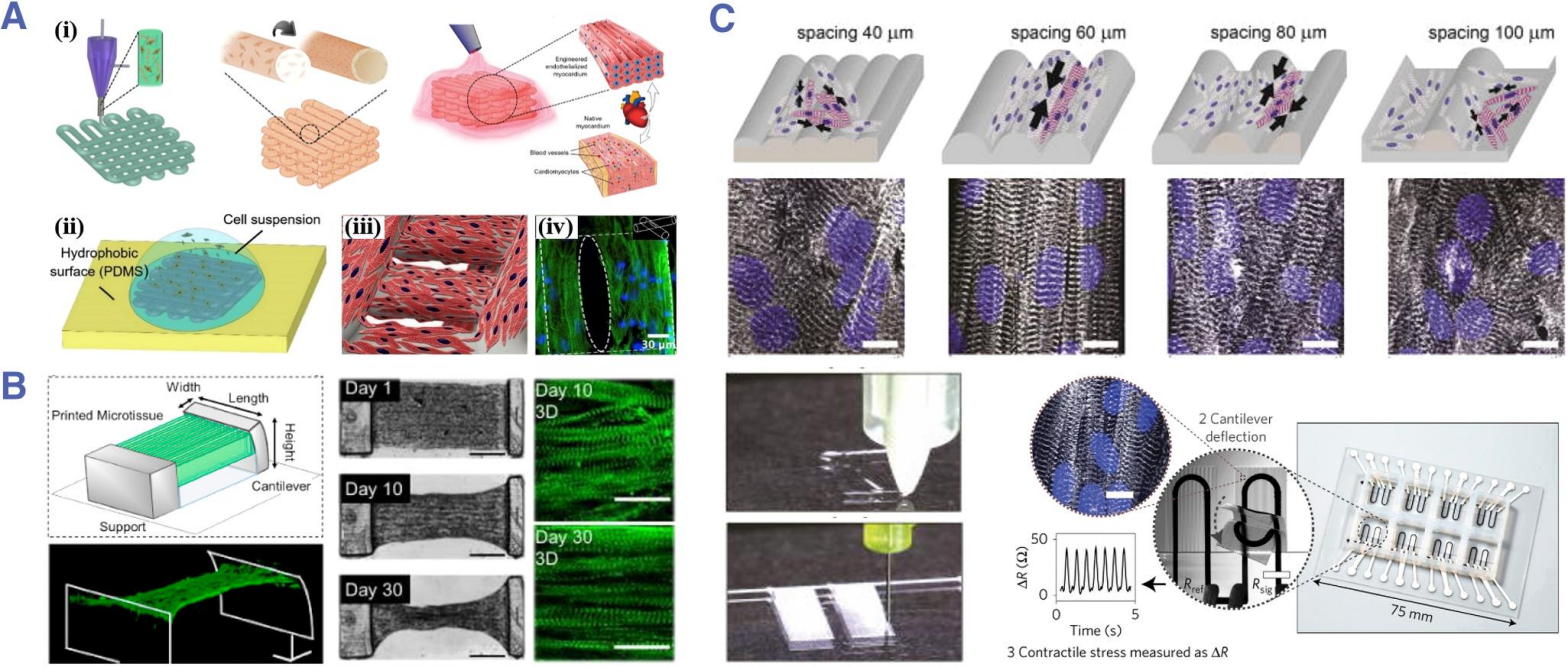
(A) Fabricating procedure and characterization of endothelialized myocardium using the 3D bioprinting strategy. Reproduced with permission.^58^ Copyright 2016, Elsevier. (B) Fabricating procedure and characterization of a μCOP‐printing heart tissue. Reproduced with permission.^61^ Copyright 2020, Elsevier. (C) Microgrooves guided cardiomyocytes assembled into anisotropic constructions and provided a noninvasive electrical readout of mechanical stress. Reproduced with permission.^62^ Copyright 2017, Springer Nature.

3D printing is recently recognized as an approach with tremendous potential for producing geometrical specified constructions, considerably enhancing their physiological significance thru engineering resembling of native organs.^30^, ^52^, ^53^, ^54^ This fabrication method enables the modulation of certain factors, including heterogeneity, construct size, matrix stiffness, and cell density, which promotes the management and development of additional complexity. Consequently, the 3D printing technique has been applied in the development of diverse tissue scaffolds to fully mimic the target tissue structure in vitro. The majority of physiological tissues are anisotropic, especially cardiomyocytes of hearts are arranged unidirectionally throughout the tissue thickness.^55^, ^56^, ^57^ Zhang et al. presented a novel hybrid strategy utilizing 3D printing technology to engineer endothelialized myocardial tissues (Figure 3A).^58^ The printed microfibrous lattices involved directly encapsulating endothelial cells to receive a layer of fused endothelium, while seeding cardiomyocytes and encouraging the creation of the myocardium with autonomous and synchronized contraction. In contrast to normal extrusion‐based 3D printing that involves one‐dimensional raster scanning of 3D objects, microscale continuous optical printing (μCOP) displays a 2D graphic into a specific volume of prepolymer, linking stage motion with changing digital mask, presenting superior *z* resolution and higher printing rate.^59^, ^60^ Chen encapsulated cardiomyocytes in μCOP‐patterned methacrylated gelatin (GelMA) construction (Figure 3B).^61^ Encapsulated cardiomyocytes successfully aligned the specified microstructure and exhibited morphological and myofibril orientation phenotypes. Parker et al. introduced a facile route for fabricating a novel class of constructed cardiac micro‐physiological instruments (Figure 3C).^62^ Specifically, they created six effective inks comprised high‐conductive, piezoresistive and compatible flexible substances, enabling the incorporation of soft tension sensors into microstructures and guiding the self‐assembling process of physiologically replicated laminated heart tissue. The authors proved effectively that these implanted sensors gave a noninvasive, electrical readout of mechanical stress. The built‐in sensors sped up data collection and allowed permanent operational investigations, opening up new paths for in vitro tissue engineering, drug screening, and toxicity research.

### 2.3 Template molding

**FIGURE 4:**
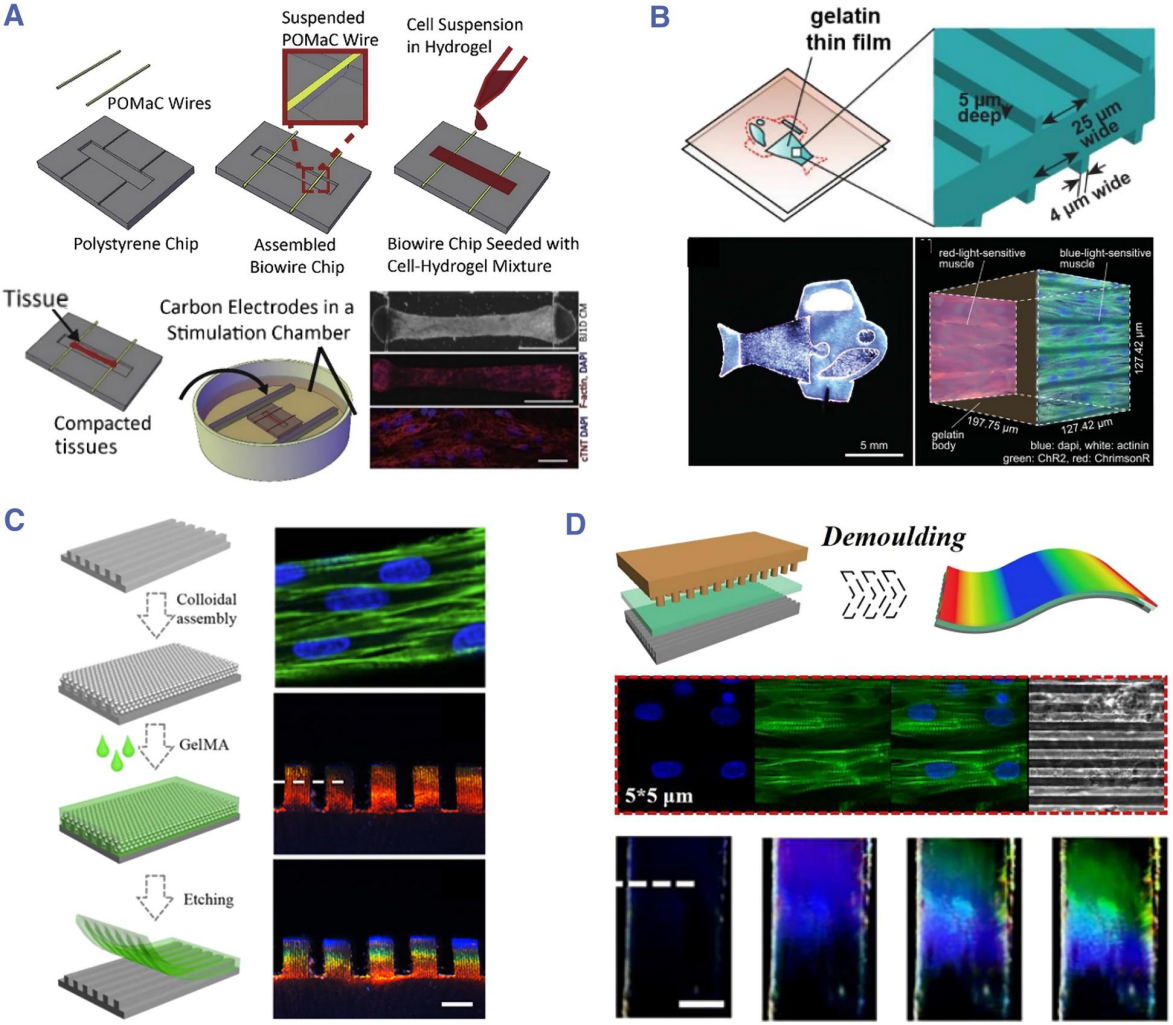
(A) Biowire II platform made up of an array of microwells printed on polystyrene plates. Reproduced with permission.^67^ Copyright 2019, Elsevier. (B) Double‐sided micro‐molded gelatin thin film containing cardiomyocytes. Reproduced with permission.^68^ Copyright 2022, The Authors, published by American Association for the Advancement of Science. (C) Fabricating procedure and optical properties of microgroove‐patterned structural color hydrogel films. Reproduced with permission.^71^ Copyright 2018, The Authors, published by American Association for the Advancement of Science. (D) Nanoimprinting process and characterization of a structural color film. Reproduced with permission.^72^ Copyright 2021, Elsevier.

Template molding has emerged as a potential high‐throughput programming technique that uses a preset pattern (master mold) to replicate the polymeric substance (target). This method depends on the idea that the structure of the required materials is retained upon removal from the mold. Common mold processing technologies include soft lithography, hot pressing, and nanoimprinting.^63^, ^64^, ^65^, ^66^ Radisic et al. designed a “Biowire” system made up of an array of microwells printed on polystyrene plates (Figure 4A).^67^ Two elastic wires made of poly (octamethylene maleate (anhydride) citrate) (POMaC) polymers were pasted along either end of the microwells. Dissociated cardiac cells were placed in a collagen hydrogel at an intensity of 5.5 × 10^7^ cells/mL. And then the suspension was seeded into microwells. The cells were “compacted” during the next week, resulting in the formation of cylindrical trabecular strips (named Biowires II), which were suspended in microwells while mechanically linked to the POMaC wires. The Biowire II platform enabled the construction of physiologically distinctive heart tissue, as well as the simultaneous assessment of force and calcium transients. Apart from simple cylindrical trabecular strips, Parker et al. created a muscular layer composite by incorporating hydrogel‐based muscular films (Figure 4B).^68^ A gel and crosslinker compound was sandwiched between two polydimethylsiloxane grooved stamps to produce the dual‐sided micro‐molded gelatin film. Cardiomyocytes were then seeded bilaterally onto the micro‐molded gelatin, enabling them to self‐assemble into laminar and anisotropic muscles, simulating the characteristics of the ventricular myocardium.

In addition, materials derived from natural creatures have undergone extensive research to utilize in synthetic manufacturing.^69^, ^70^ Dynamic iridescence has aroused great interests due to the present numerous stunning specimens. Inspired by the structural color regulation mechanism of chameleons, Fu et al. employed microgroove‐patterned silicon wafers for the templates self‐assembling and hydrogel replicating (Figure 4C).^71^ The resulting hydrogel films were endowed with surface microgroove structures and angle‐dependent structural color properties. Owing to the micromorphology and excellent biocompatibility of the hydrogel film, the formed cardiomyocytes succeeded in restoring their autonomous beating with ordered cell orientations and increased contractile functioning. Since the beating operations of cardiomyocytes were followed by cell contraction and elongation, the hydrogel film underwent a corresponding cycle of structural color changes. Afterward, this research group employed nanoimprinting technology to fabricate a film with anisotropic microgroove and 2D photonic crystal structures (Figure 4D).^72^ This film was endowed with outstanding orientation induction function as well as cardiomyocyte dynamic display. Thus, these structural color materials could give inherent color‐sensing signals to modulate systemic issues in future designed tissue.

### 2.4 Decellularized ECM

Decellularized ECM scaffolds hold great promise toward tissue transplantation and regenerative medicine.^73^, ^74^, ^75^, ^76^ The objective for employing decellularized ECM is to imitate the natural physiological microenvironment, which is essential for regaining equilibrium in cells through affecting cell functions and behaviors. The decellularized ECM scaffolds are predicted to restore tissue operation through boosting cell adhesion, differentiation, and proliferation, thus offering an enduring therapeutic solution for tissue repair. Several decellularization strategies have been developed to eliminate potentially harmful hosting antigens and cells while retaining the original decellularized ECM structure and fundamental physiological parts: physical procedures (agitation, pressure, ultrasonic treatment, freeze‐thawing, and mechanics strokes), enzyme therapy (protease or nuclease), and chemical reagents (acids, detergents, alkalis, chelating chemicals, organic solvents, hypertonic solutions.^75^, ^76^

**FIGURE 5:**
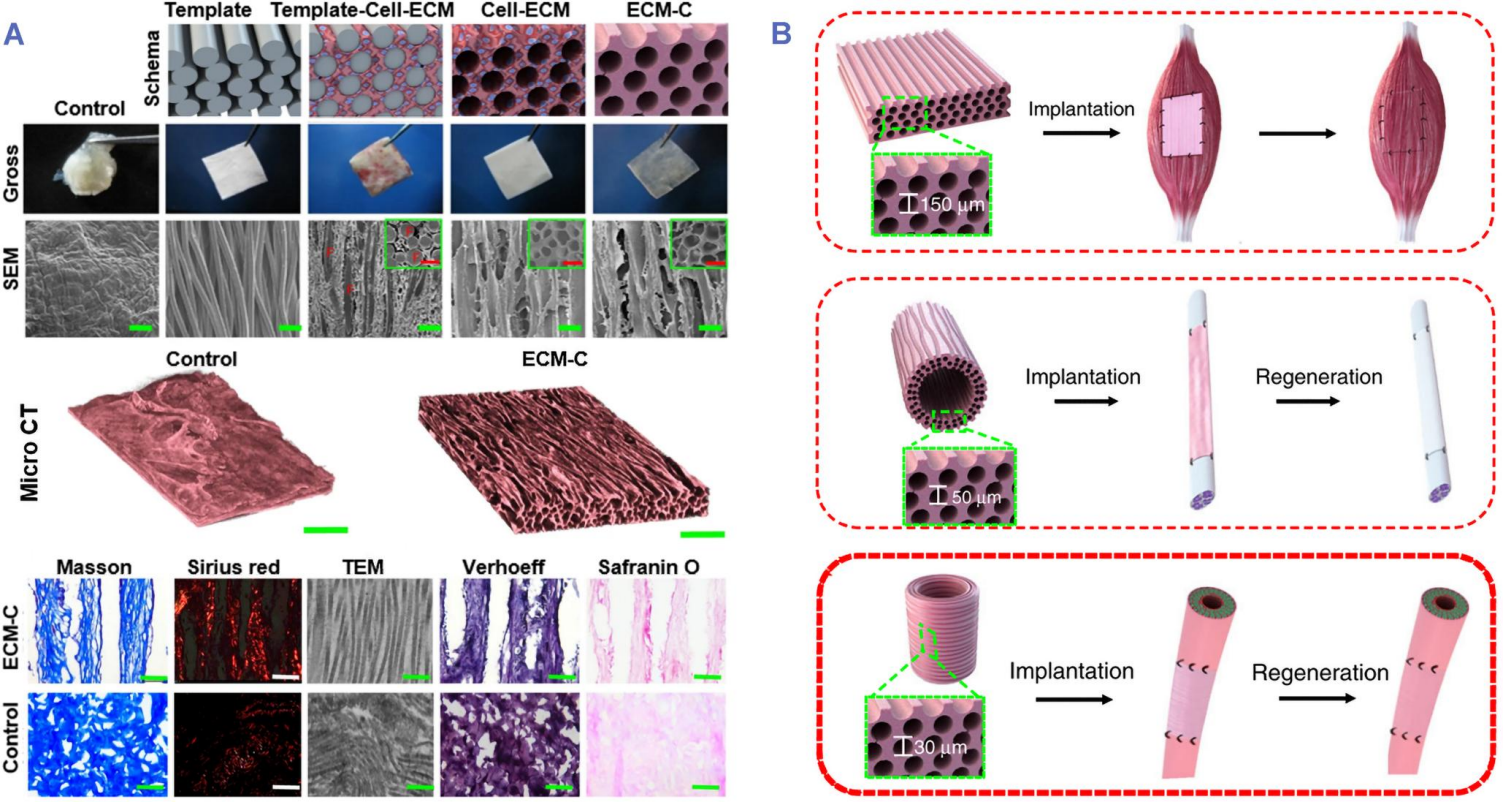
(A) Fabrication and characterization of ECM scaffolds. (B) The versatility of ECM scaffolds in guided regeneration. Reproduced under terms of the CC‐BY license.^31^ Copyright 2019, The Authors, published by Springer Nature.

Mercuri et al. were the initial group to successfully decellularize the nucleus pulposus (NP) using a combined approach of detergents, ultrasonication, and enzymes to construct a cell‐free NP framework.^77^ Zhou et al. used genipin to cross‐link the sulfate glycosaminoglycan lost during decellularization and created an injectable NP‐based cell delivery system (NPCS) with components identical to the original NP.^78^ In vitro testing demonstrated that the NPCS was biocompatible and capable of inducing NP‐like differentiation of adipose‐derived stem cells. In vivo implanting tests revealed that the NPCS could partially repair the degraded NP in a well‐established animal model. This novel bionic scaffold exhibited potential to treat intervertebral disc deterioration. Nevertheless, the absence of organized porosity construction prevents cells from receiving guidance cues in directed migrating and spatial organization, therefore limiting the morphofunctional integration of instructed tissues. Zhu et al. obtained a new decellularized ECM scaffold with parallel microchannels by implanting sacrificial templates subcutaneously, removing the templates, and decellularizing them (Figure 5).^31^ This work explored the influence of the generated scaffolds on cell migration and directed tissue development with favorable vascularization and immunomodulation. Zhu et al. demonstrated the plasticity and adaptability of such scaffolds by rebuilding functionally integrated innervated and vascularized neo‐muscles and pulsating neo‐arteries. The technique offered the prospect to produce activated biomedical materials for tissue construction and medical regeneration.

### 2.5 Self‐assembly

**FIGURE 6:**
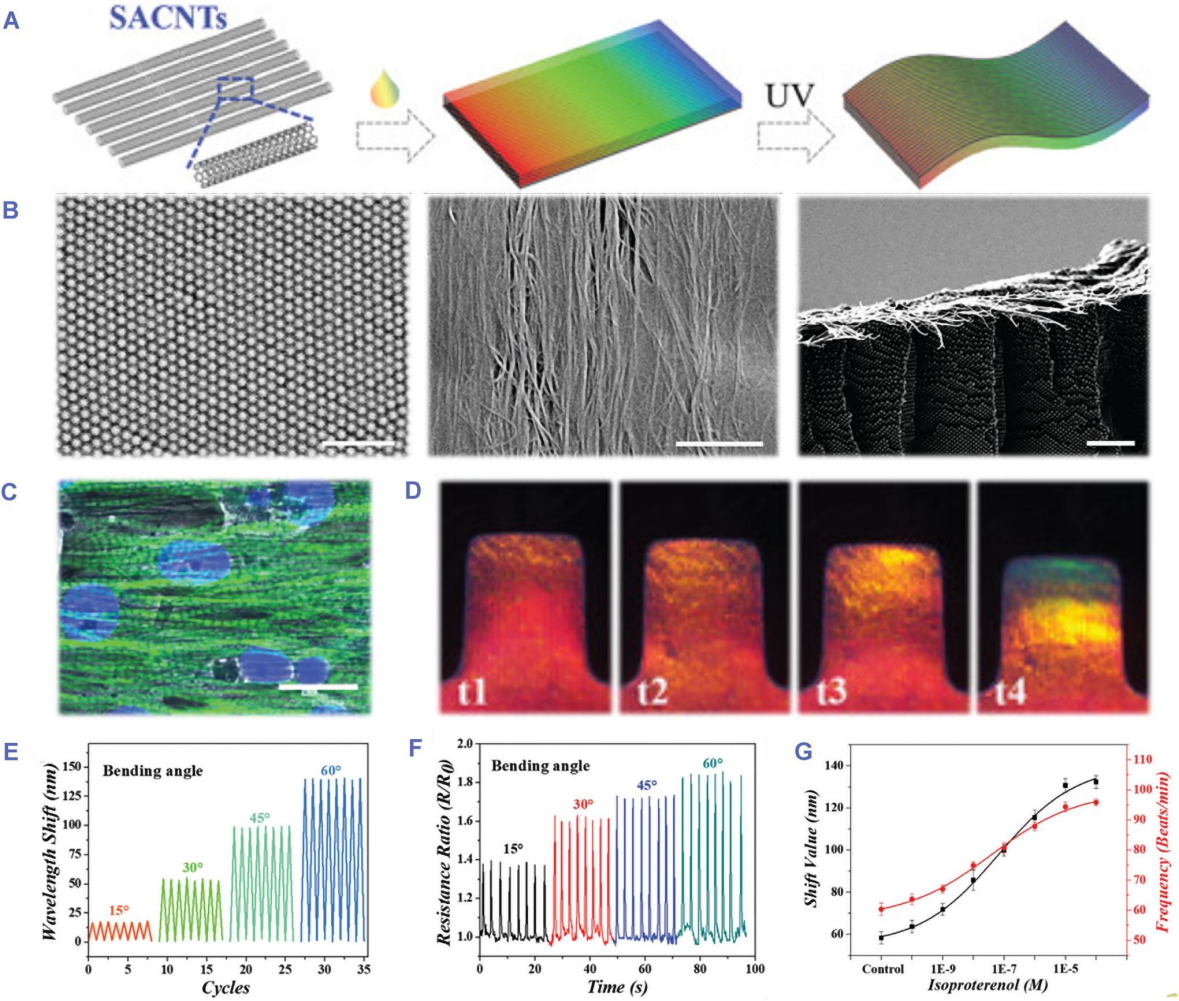
(A) Fabrication of an anisotropic electroconductive hydrogel. (B) Microtopography of the structural color hydrogel. (C) Confocal image of cardiomyocytes on the hydrogel with SACNTs. (D–G) Optical properties and electrical properties of the hydrogel. Reproduced under terms of the CC‐BY license.^83^ Copyright 2022, The Authors, published by John Wiley and Sons.

Self‐assembly has emerged as an efficient technique for spontaneously generating a scheduled, highly ordered structure with no external influences.^79^, ^80^ Given its inherent superiority, self‐assembly is considered as a potential strategy for constructing nanomaterials. Colloidal self‐assembly has achieved substantial development according to the superior properties of colloidal substrates.^81^, ^82^ Sun et al. created an anisotropic electroconductive structured color hydrogel (Figure 6).^83^ In detail, the surface of negative‐charged silica nanoparticles was produced and dispersed in acrylamide (AAm) prepolymer. The charged silica nanoparticles then assembled into nonclose packed arrays as a result of minimal energy arrangement and charge repulsion. Because of the organized nanoparticles, the silica nanoparticle‐doped hydrogel was endowed with a gorgeous color. Furthermore, to create freestanding hydrogel, super aligned carbon nanotube sheets (SACNTs) lined up on the glass were infiltrated by a bio‐compatible prepolymer for pre‐polymerizing and impregnated with AAm dispersion liquid. After ultraviolet (UV) irradiation, the hydrogel was obtained with a SACNT layer on the surface and a colloidal array in the bottom. It was found that the SACNT structure could properly promote cardiomyocyte alignment, and that SACNT conductivity might facilitate cardiomyocyte rhythmic beating. Furthermore, the structural color and electrical resistance of the hydrogel demonstrated a rapid and apparent feedback signal to cardiomyocyte beating. These characteristics suggested that the colorful hydrogels were extremely beneficial in sensing chips.

### 2.6 Other methods

**FIGURE 7:**
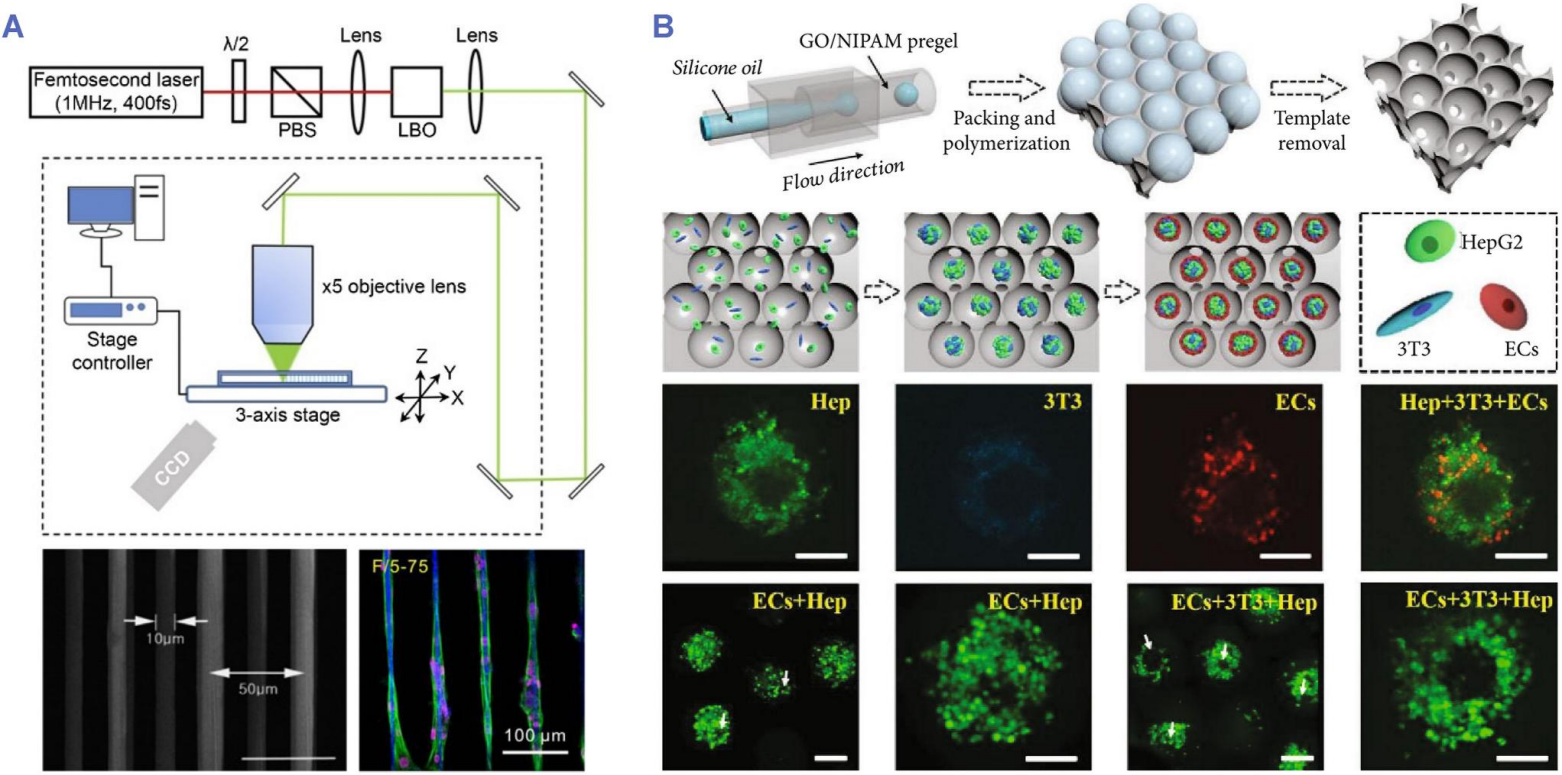
(A) Bioinspired cardiac tissue model with the TPIP technique. Reproduced with permission.^87^ Copyright 2014, Elsevier. (B) Multifunctional scaffolds obtained by combining microfluidic and template molding techniques and their characterization. Reproduced under terms of the CC‐BY license.^88^ Copyright 2019, The Authors, published by American Association for the Advancement of Science.

Much effort and focus has been put into employing advanced technologies to foster the technology of manufacturing biomedical materials with microcosmic architecture. Electrospinning is a versatile and adaptable technology for creating microcosmic fibers with varying morphologies and diameters from suspensions, solutions, or melts using electrodynamic power.^84^ The 3D electrospun scaffolds with aligned nanofibers can mimic the orientation of ECM in the heart, facilitating the self‐organization of cells into the anisotropic structure. Nevertheless, electrospinning usually results in uncontrollable nanopores and fails to directly inject cells into the matrix to generate 3D cell structures. More improved manufacturing technologies are required to construct scaffolds with precisely specified micro‐nano characteristics that enable the investigation of human stem cell function. Two‐photon initiated polymerization (TPIP) refers to a laser writing technique, which is generally restrained to treat photoresist near the laser's focal volume, allowing the fabrication of programmable 3D models with a geometric precision close to 100 nm.^85^, ^86^ Ma et al. used the TPIP approach to develop a cardiac tissue with a set of 3D filament matrix that strictly controlled cardiomyocyte alignment and modified cell‐mediated mechanical environments (Figure 7A).^87^ Further, by combining various procedures and components, it is possible to fabricate materials with intricate architectures and various functions. Shao et al. presented a versatile scaffold by using graphene oxide and N‐isopropylacrylamide mixture (Figure 7B).^88^ A scaffold enrichment process with multiple cell types could result in the biological development of diverse physiologic and pathologic models for organ‐level physiology and in vitro pharmaceutical screening. Beyond these accomplishments, various attempts have been currently conducted to overcome the complexities and constraints of these systems. Additional investigation intends to produce simple technologies, which may displace present complicated technology with simple, dependable, versatile, and efficient methods, eliminate operating time, and aid in mass manufacturing as well as industrial purposes.

## 3 CONSTRUCTION OF CARDIAC FIBROSIS

The cardiac fibrosis procedure is regarded as a series of related actions for healing processes that eventually develop into persistent inflammatory responses and lead to fibrosis. Due to the production of pro‐fibrotic and inflammatory cytokines, and increased mechanical strain, heart damage causes fast activation and proliferation of nonactivated stromal cells as well as mature cardiac fibroblasts.^89^, ^90^ Particularly, long‐lasting activation of cardiac fibroblasts under extended detrimental stress conditions, in particular, causes persistent formation and deposition of stiff ECM, resulting in heart failure, diastolic failure, and elevated vulnerability to fatal heart rhythm disturbances.^91^ The current challenge of cardiac fibrosis directly highlights our poor understanding of complicated pathological procedures and the necessity to develop novel cardiovascular drugs.

However, conventional 2D cultures without natural physiologic environments are inadequate for a comprehensive understanding of cardiac fibrosis. In addition, a large proportion of compounds exhibit potential in preclinical animal studies but lack efficacy in human clinical trials.^92^ Therefore, there is an urgent need for well‐defined advanced models to improve the understanding of the fibrotic heart. In this chapter, we focus on the establishment of in vitro cardiac fibrosis through representative examples and discuss the latest advancements and their corresponding applications.

### 3.1 Dynamic stretching

The cardiac system serves as a highly dynamic system with synchronized contraction and relaxation behaviors. The fundamental biomechanical aspect of the heart at the organ level is continuous stretching caused by the beating. In addition, continuous stretch at the cellular level is caused by the regular rhythm of cardiomyocytes and, to a lesser extent, by the contraction of myofibroblasts. Current research suggests that immoderate stretching could act as a significant stimulant for myocardial injury and continuing activation of myofibroblasts, ultimately leading to the development of fibrosis.^93^, ^94^, ^95^ McCain et al. imitated mechanical overexertion by circularly stretching designed layered cardiovascular tissues on an elastic chip.^96^ Periodic stretching was found to activate indicators of abnormal heart hypertrophy, change myocyte morphology and filament orientation, modify calcium transient, and diminish stress production.

**FIGURE 8:**
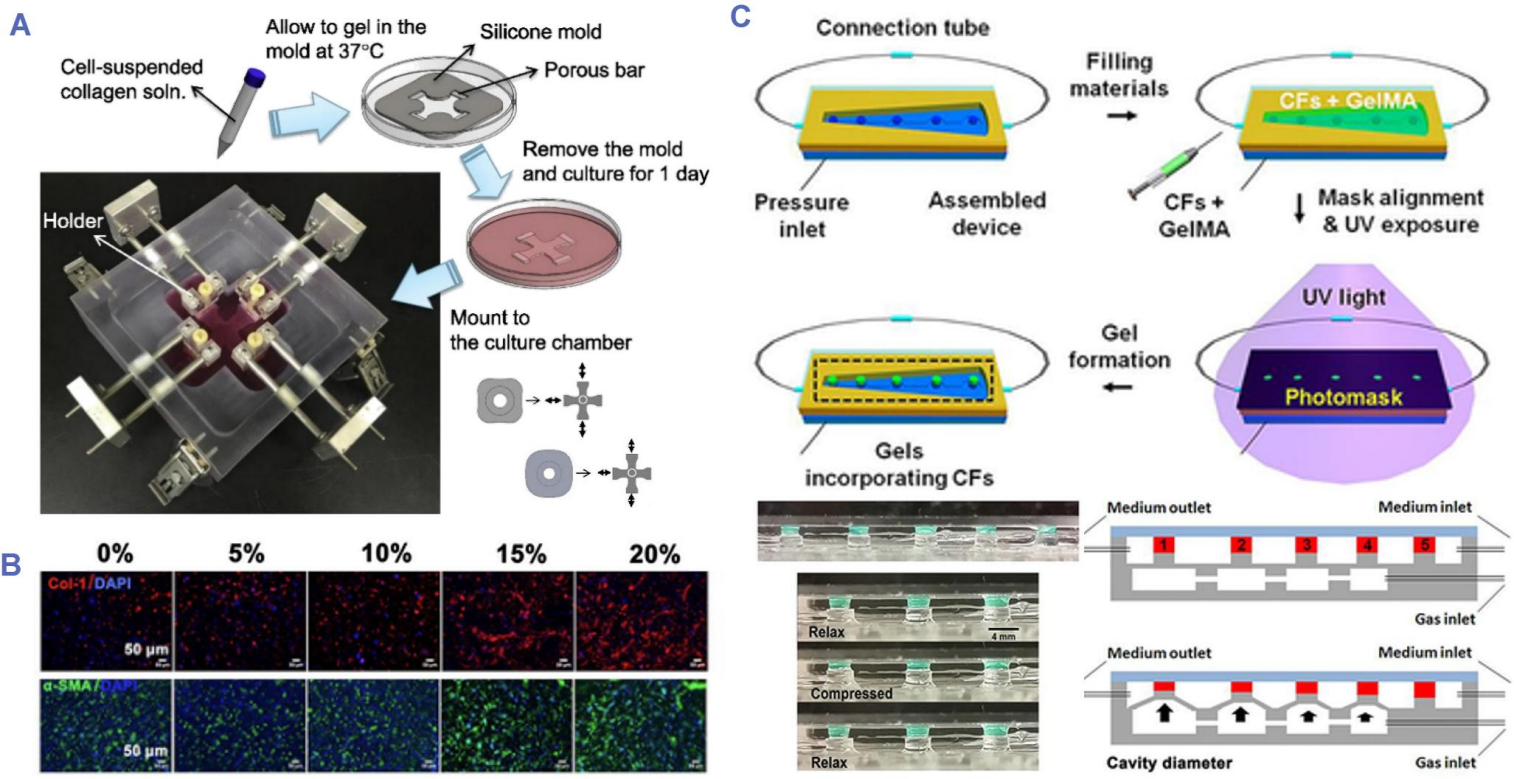
(A) Dynamic biaxial culture system providing cyclic stretching of fibroblast‐loaded gels. Reproduced with permission.^99^ Copyright 2018, Elsevier. (B, C) Cardiac fibrosis on a chip employing adjustable cyclic compression on the hydrogel containing cardiac fibroblasts. Reproduced with permission.^95^ Copyright 2019, John Wiley and Sons.

As a key component of organ tissues, fibroblasts are subjected to a range of dynamic pressures.^97^, ^98^ Researchers have created a multitude of systems or devices to show how the applied forces affect the behavior of fibroblasts. Lee et al. created a dynamic biaxial culture system capable of cyclic stretching of fibroblasts‐loaded gels (Figure 8A).^99^ It was discovered that fibroblasts under continuous bilateral stretching exhibited a lengthened spindle‐like shape with higher alpha smooth muscle actin (α‐SMA) expression. In addition, cell culture experiments were conducted on various stretchable planes such as silicone membranes, which were found to have significantly increased scarred reactions such as fibroblast growth, collagen production, and network metalloproteinases.^100^, ^101^ Kong et al. investigated the association between motion and cardiac fibroblast growth using cycled compressing with gradient intensity and configurable rate (Figures 8B, C).^95^ They found that cyclic compression caused the proliferation and phenotypic transformation of cardiac fibroblasts. However, traditional single‐cell culture platforms failed to encapsulate higher dimensions as well as higher‐order intercellular interactions. This prompted the need for a human bionics in vitro platform to study the progress of 3D fibrotic remodeling.

### 3.2 Pro‐fibrotic molecules

**FIGURE 9:**
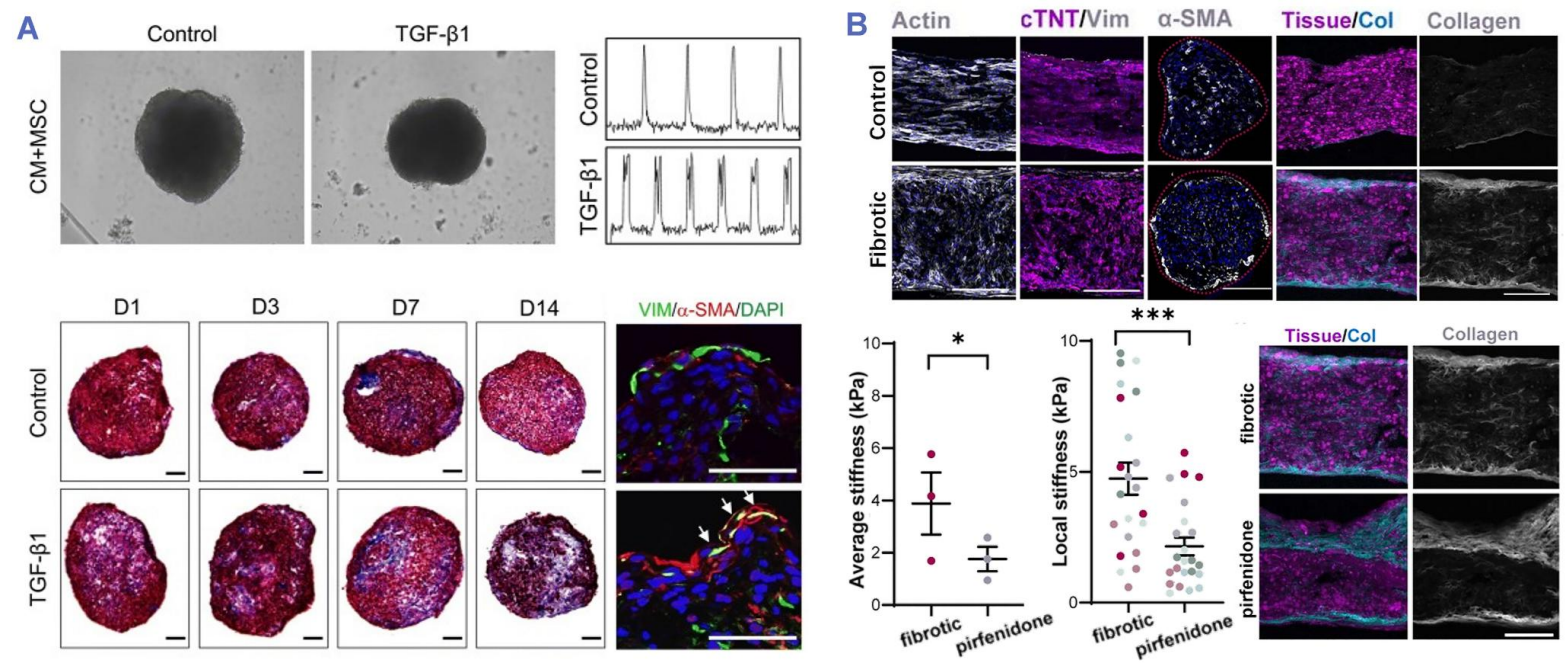
(A) Generalization of phenotype associated with TGF‐β1‐induced cardiac fibrosis tissue. Reproduced under terms of the CC‐BY license.^15^ Copyright 2019, The Authors, published by Springer Nature. (B) The fibrosis features and drug screening function of the hCF‐on‐a‐chip. Reproduced with permission.^105^ Copyright 2020, Elsevier.

Transforming growth factor‐β1 (TGF‐β1) serves as a key regulating cytokine responsible for tissue healing, and its continuous synthesis in numerous tissues provides the cornerstone of fibrosis development.^102^, ^103^, ^104^ Extensive evidence from animal models and human biopsies supports a central role for TGF‐β1 in tissue fibrosis. Sadeghi et al. prepared a 3D hydrogel platform with physiological stiffness encapsulating newborn rat cardiac cells and indicated the pro‐fibrotic effects of induced cardiac fibroblast activation.^9^ However, the main limitation was the use of rat cardiac cells, which failed to predict human cell response. Particularly, cardiomyocytes generated from human pluripotent stem cells (hPSCs), like human induced pluripotent stem cells (hiPSCs) and human embryonic stem cells (hESCs), are currently viewed as the most suitable cellular source for in vitro heart modeling. Lee et al. developed a 3D cardiac microstructure comprised of mesenchymal stem cells (MSCs) and hESC‐CMs (Figure 9A).^15^ MSCs could differentiate toward myofibroblasts upon TGF‐β1 stimulation, resulting in a 3D cardiac microstructure exhibiting fibrotic features. Mastikhina et al. further developed a human cardiac fibrosis (hCF‐on‐a‐chip) using computer numerical control milling technology and inoculated a fibrin gel doped with hiPSCs‐derived cardiomyocytes and human cardiac fibroblasts activated with TGF‐β1 into the microwells (Figure 9B).^105^ After sustained TGF‐β1 stimulation, hCF‐on‐a‐chip showed the hallmark features of cardiac fibrosis and associated heart failure. In addition, they found that colesartan and carvidiol significantly reduced BNP secretion in the hCF‐on‐a‐chip. Besides, pirfenidone, used to treat idiopathic pulmonary fibrosis, also significantly reduced tissue stiffness and BNP secretion, and improved microRNA profiles in tissues with significant loss of functional capacity. These findings suggested that engineered heart tissue might serve as an effective model for investigating fibrous modifications in the heart and that they might facilitate the creation of improved biologically feasible preclinical drug screening systems.

### 3.3 Variation of cellular composition

**FIGURE 10:**
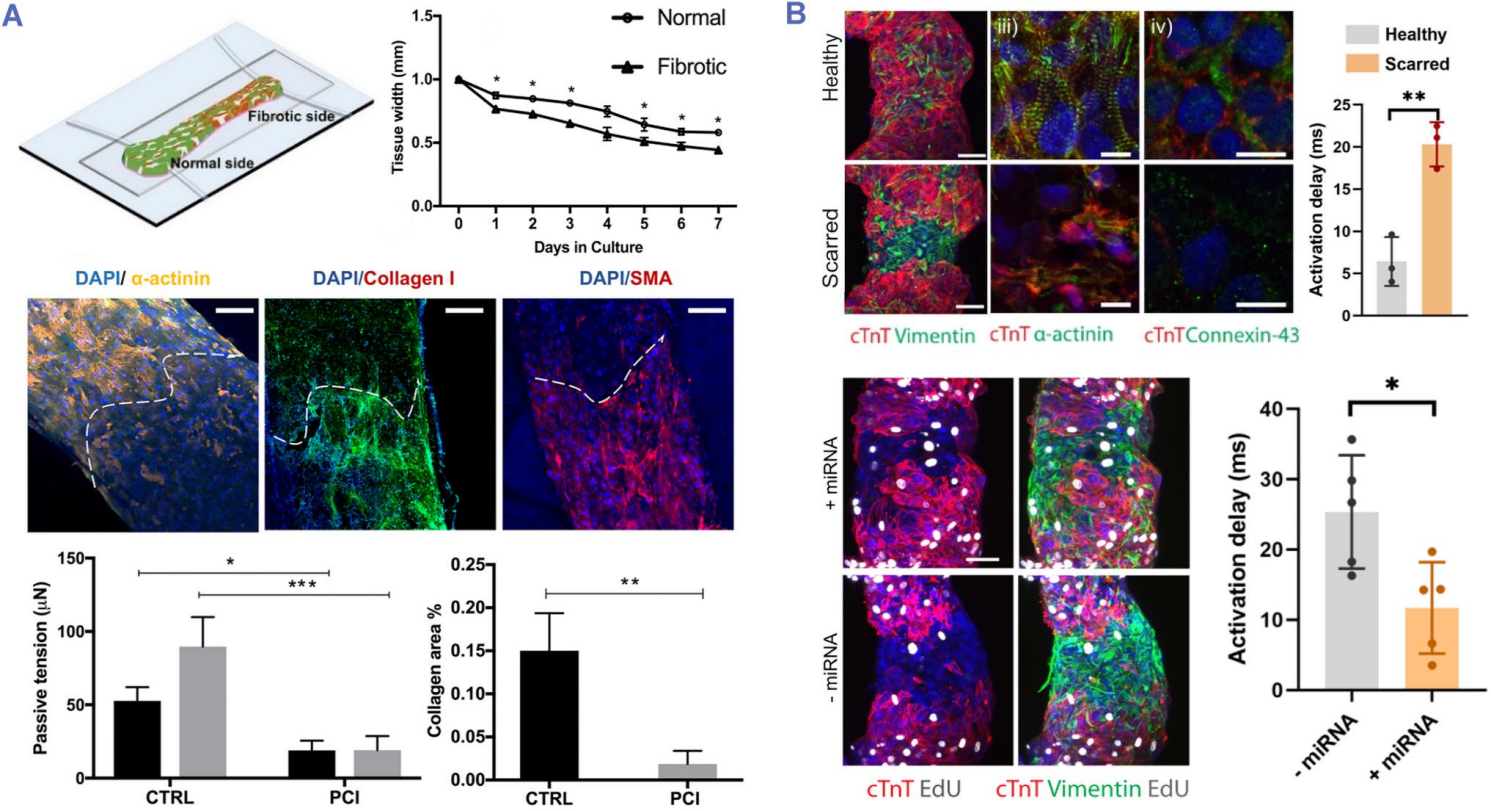
(A) Construction and evaluation of the fibrotic myocardium tissue. Reproduced with permission.^110^ Copyright 2019, American Chemical Society. (B) 3D bioprinting cardiac microtissues for disease modeling and evaluation of miRNA therapy. Reproduced under terms of the CC‐BY license.^111^ Copyright 2021, The Authors, published by Springer Nature.

As an important role in maintaining tissue homeostasis, cardiac fibroblasts assist ECM angiogenesis and homeostasis, and operate on myocardial cells, blood vessels, and immune cells via paracrine activities.^91^, ^106^, ^107^ Nevertheless, overpopulated cardiac fibroblasts lead to augmented collagen accumulation and tissue rigidity, which triggers the myofibroblast differentiation and exacerbates the collagen deposition, leading to fibrosis.^4^, ^5^ Therefore, researchers achieved in vitro cardiac fibrosis by simulating a high proportion of cardiac fibroblasts in diseased hearts.^108^, ^109^ For example, Wang et al. simulated fibrotic myocardium by reducing the relative proportions of cardiomyocytes and cardiac fibroblasts (Figure 10A).^110^ In detail, hiPSC‐derived CMs and cardiac fibroblasts (75% or 25%) were dispersed in fibrin/matrigel hydrogel, and electrical conditioning was used to improve tissue function and maturation. In a mature heart, tissue with a high proportion of fibroblasts demonstrated mechanical and physiological hallmarks of fibrous cardiology. Following spatial patterning of diverse microtissues, they created a heteropolar simulation comprising both sections and constructed a disease system that replicated scarring lesions incorporation into ordinary hearts. They also explored the effect of furin inhibitors using the model platform and validated its potential for drug testing applications. Further, Daly et al. developed heart disease models that emulated the formation of scars after myocardial infarction by bioprinting the microtissues with spatially controlled cardiomyocyte and fibroblast cell ratios (Figure 10B).^111^ The microtissue, which consisted of a high proportion of cardiac fibroblasts, successfully replicated the pathological features of cardiac fibrosis, including reduced systolic output and asynchronous electrophysiological signals. Additionally, this microtissue was utilized to explore miRNA therapy, demonstrating its potential in drug screening.

### 3.4 Other methods

**FIGURE 11:**
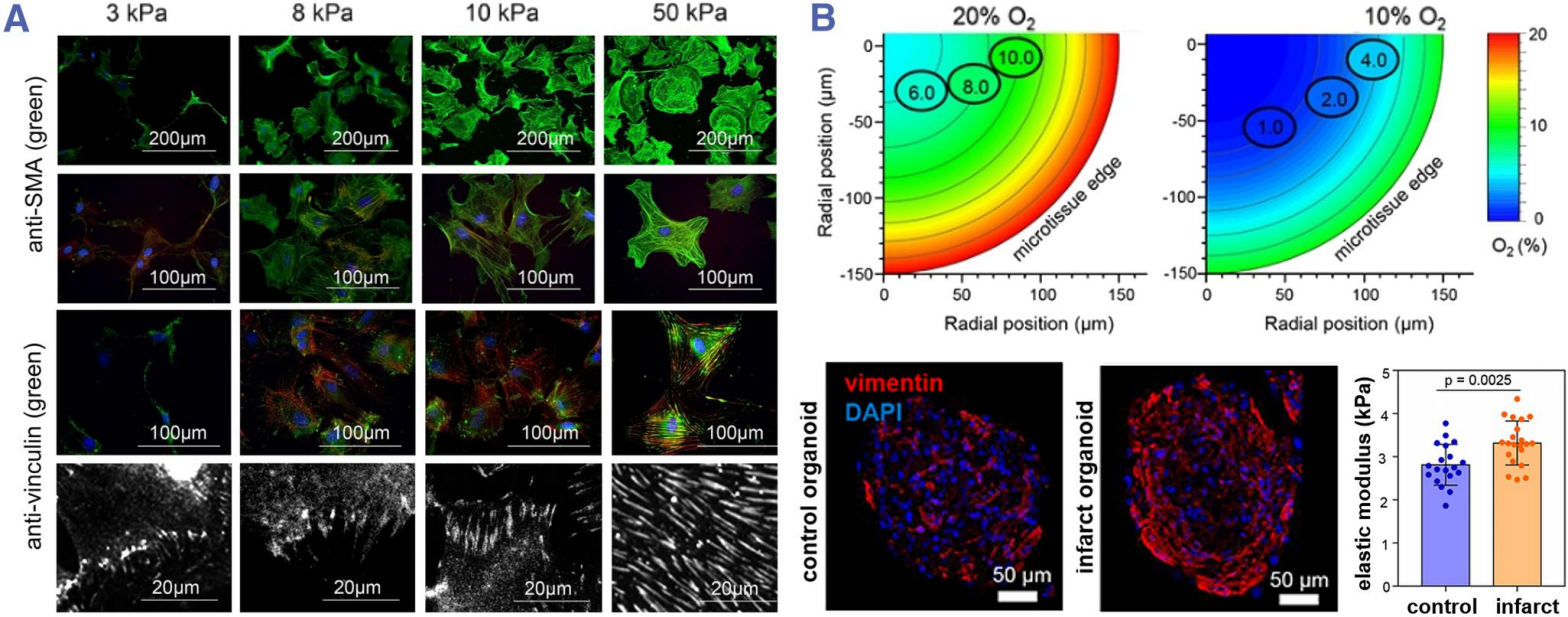
(A) Maintaining a quiescent cardiac fibroblast phenotype by controlling substrate stiffness. Reproduced with permission.^116^ Copyright 2017, The Authors, published by the American Society for Cell Biology. (B) Construction of hypoxic‐induced myocardial infarction in vitro and characterization of pathological fibrosis response. Reproduced with permission.^14^ Copyright 2020, The Authors, published by Springer Nature.

Increased mechanical stiffness may affect cellular behavior via receptors and signal transduction systems.^112^, ^113^, ^114^, ^115^ Researchers investigated the effect of matrix stiffness on myofibroblast activation by cultivating cardiac fibroblasts with stiffness‐tunable gels. The results showed that high‐stiffness substrates mimicked fibrotic heart tissue and promoted the differentiation of cardiac fibroblasts (Figure 11A).^116^, ^117^ Researchers found that increased stiffness promoted integrin‐trapped latent TGF‐β on myofibroblast membranes, which then activated TGF‐β.^118^ In addition, increased stiffness also promoted TGF‐β receptor activity, which induced myofibroblasts to generate ECM collagen and protein.^119^, ^120^ This process exacerbated the stiffness of the injured myocardium, particularly during pathological fibrosis following myocardial injury, thereby impeding both physiologic heart maturation and cardiac fibrosis degeneration.

In addition, cardiac microtissue has also been used to simulate cardiac fibrosis caused by infarct injury.^121^ Richards et al. outlined the “distal infarct border region” of the infarcted heart through hypoxia and chronic adrenergic stimulation of 3D microstructures (Figure 11B).^14^ Their findings revealed that this “infarct‐like organ” expressed multiple fibrosis‐related genes, presented myofibroblast‐like cells, and demonstrated a boost in microstructural rigidity. They also tested heart failure drugs at the tissue level using this model. In addition, macrophages are present in almost all vertebrate tissues, and they maintain immunity by phagocytosing microorganisms, removing senescent cells, and repairing and regulating organ function for homeostasis in vivo.^122^, ^123^ During myocardial infarction, macrophages in the heart exhibit local proliferation and recruitment responses, which secrete fibrosis‐promoting factors such as angiotensin II, TGF‐β, and platelet‐derived growth factor that exacerbate the progression of heart failure.^124^, ^125^, ^126^ Given the significance of macrophages in the cardiac fibrosis process, a cardiac fibrosis model using macrophages is deemed desirable.

## 4 CONCLUSIONS

In summary, this review outlines the construction of cardiac fibrosis. We first present biomedical materials and discuss the corresponding fabrication methods. In particular, we introduce novel combinations between multifunctional materials and special structures, such as anisotropic surface topologies and various sensing materials, and discuss the applications and advantages in tissue engineering and in situ detection. Then, based on 3D multi‐cell cultures, we summarize emerging in vitro models of cardiac fibrosis, with particular emphasis on the methods of fibrosis induction and the detailed applications of drug screening. These features provide a novel strategy toward improving our comprehension of cardiac diseases and facilitating sensitive and high‐throughput drug screening.

Biomedical materials based on micro‐nano technologies have aided in the construction of in vitro model systems and facilitated the progress of in vitro monitoring systems based on sensing signals. However, current fibrotic models based on biomaterial platforms are excessively simplistic and lack the simulation of in vivo tissue structures. With advances in microfabrication technologies, the development of simple alternative manufacturing and synthesis methods should be explored in an effort to increase the accessibility of these systems to their intended users. Besides, non‐invasive measurements of tissue electrical impedance can be used to assess tissue scar formation. For instance, scarred myocardial tissue exhibits lower impedance compared with healthy myocardium. Impedance variations between tissues are important and can be used to identify different tissue layers or to distinguish malignant from benign tumors. To our knowledge, biomaterials with modifiable electrical impedance have not been used in fibrosis or mechanobiology studies. These indicate that such biomaterials are important for understanding the role of bioelectricity in fibrosis and wound healing.

Furthermore, cells from traditional in vitro cardiac models fail to fully represent human drug response, such as those from mammary rats. Notably, the advent of novel organoid technologies has enabled the development of patient‐specific in vitro cardiac models. In particular, given the patient‐specific nature of organoids, individual patient‐based precision drug delivery systems are widely expected. While various multicellular in vitro co‐culture systems achieve optimum organ and tissue interaction, static growth conditions may restrict the maintenance of cardiac structure and function, and further affect the accuracy of drug evaluation. A specially designed microfluidic system can represent an appropriate option for the constant provision of nutrition and disposal of waste products in a timely manner. Therefore, microfluidic system‐based organ‐on‐a‐chip shows great promise in the exploration of simulation of heart diseases. A multipronged approach to optimize drug screening studies is necessary in the future to test potential drugs and design effective therapeutic strategies. We believe that the above‐mentioned challenges of cardiac fibrosis could motivate the development of basic and applied science and contribute significantly to advancing the biomedical applications and the clinical translation of these technologies.

## AUTHOR CONTRIBUTIONS

Lingyun Sun conceived the study. Yixuan Shang wrote the manuscript. Jingjing Gan, Yuzhi Yang, and Rui Liu helped revise the manuscript. All authors have read and approved the final manuscript.

## CONFLICT OF INTEREST STATEMENT

All authors declare that there are no competing interests.
